# Impact of Denosumab Adherence on Renal Function and Mortality Rates in Type 2 Diabetes Patients With Osteoporosis

**DOI:** 10.1002/kjm2.70124

**Published:** 2025-10-09

**Authors:** Yu‐Chuan Chang, Jian‐Chih Chen, Sung‐Yen Lin, Kun‐Der Lin, Pei‐Shan Ho, Chung‐Hwan Chen, Yin‐Chih Fu, Tien‐Ching Lee

**Affiliations:** ^1^ School of Post‐Baccalaureate Medicine, College of Medicine Kaohsiung Medical University Kaohsiung Taiwan; ^2^ Department of Orthopedics, College of Medicine Kaohsiung Medical University Kaohsiung Taiwan; ^3^ Department of Orthopedics Kaohsiung Medical University Hospital, Kaohsiung Medical University Kaohsiung Taiwan; ^4^ Department of Orthopedics Kaohsiung Medical University Gangshan Hospital Kaohsiung Taiwan; ^5^ Department of Orthopedics, School of Post‐Baccalaureate Medicine, College of Medicine Kaohsiung Medical University Kaohsiung Taiwan; ^6^ Regenerative Medicine and Cell Therapy Research Center Kaohsiung Medical University Kaohsiung Taiwan; ^7^ Orthopaedic Research Center, College of Medicine, Kaohsiung Medical University Hospital, Kaohsiung Medical University Kaohsiung Taiwan; ^8^ The Lin's Clinic Kaohsiung Taiwan; ^9^ Faculty of Dental Hygiene, College of Dental Medicine Kaohsiung Medical University Kaohsiung Taiwan

**Keywords:** adherence, denosumab, kidney function, osteoporosis, type 2 diabetes mellitus

## Abstract

Type 2 diabetes mellitus (T2DM) is associated with an increased risk of osteoporosis and fractures, and denosumab, a non‐kidney‐excreted antiresorptive medication, represents a viable alternative to bisphosphonates for osteoporosis treatment in patients with T2DM. This study aimed to investigate the association among denosumab adherence, renal function, and all‐cause mortality in patients with T2DM and osteoporosis. New denosumab users between 2010 and 2017 were identified from an electronic health record database, and after exclusion, 536 participants were screened and analyzed based on their 2‐year drug adherence: high adherence (HA) defined as three or four doses, and low adherence (LA) defined as one or two doses. The 1‐year average estimated glomerular filtration rate (eGFR) was calculated, and all‐cause mortality was analyzed using Kaplan–Meier curves and Cox regression models. The study included 286 and 250 subjects in the HA and LA groups, respectively, and although eGFR declined in both groups, renal function remained comparable between the groups. The all‐cause mortality rate was significantly lower in the HA group compared to the LA group (adjusted hazard ratio: 0.52, 95% confidence interval: 0.29–0.92). High denosumab adherence was found to be associated with a lower risk of all‐cause mortality among patients with T2DM and osteoporosis without significantly impacting renal function, highlighting the potential benefits of maintaining regular denosumab treatment in this high‐risk population. Nevertheless, this observational study design indicates association rather than causation, and further prospective research is warranted to validate these results and elucidate the mechanisms underlying the relationships observed in this study.

## 1 Introduction

Patients with type 2 diabetes mellitus (T2DM) are at a higher risk for osteoporosis and related fractures, particularly at the hip and the spine [1, 2, 3]. Osteoporosis affects approximately 18.3% of the global population, depicting a higher prevalence in women (23.1%) than in men (11.7%) [4]. Among individuals with T2DM, osteoporosis prevalence has risen to 37.8% and is especially high in women (44.8%) compared to men (37%) [5]. Hyperglycemia impairs the bone mineral density (BMD) by disrupting the balance between osteoblasts and osteoclasts, promoting bone resorption by increasing the levels of osteoclasts, TNF‐α, M‐CSF, and RANKL, and ameliorating the factors that support bone formation, such as Runx2, osteocalcin, osteonectin, and osteoblast proliferation [6, 7]. Microvascular complications further compromise bone health by affecting the bone microvasculature and increasing the bone marrow adiposity. Additionally, advanced glycation end‐products contribute to bone loss by inducing oxidative stress and inflammation [8].

T2DM is a leading cause of end‐stage renal disease and substantially increases the risk of cardiovascular disease [9]. The estimated glomerular filtration rate (eGFR) naturally declines after the age of 40, and poor glycemic control further accelerates this decline [10, 11]. Individuals with diabetes are nearly twice as likely to develop chronic kidney disease (CKD) compared to those without diabetes [12]. Bisphosphonates, employed as the standard therapeutic agents for osteoporosis, are predominantly excreted by the kidneys and might cause nephrotoxicity, making them unsuitable for patients with severe renal impairment [13, 14, 15].

Adherence to osteoporosis medications is critical, as poor compliance has been consistently linked to increased mortality rates in patients with osteoporosis [16, 17, 18]. Denosumab is a human monoclonal antibody that inhibits osteoclast‐mediated bone resorption and confers a distinct advantage for patients with impaired renal function because it is not excreted by the kidneys and does not require dose adjustment [19, 20]. Multiple clinical studies and real‐world data have demonstrated that denosumab is both safe and effective in patients with mild to moderate CKD without any evidence of adverse effects on renal function [21, 22]. Furthermore, recent large‐scale cohort studies and meta‐analyses have shown that denosumab use is associated with a reduced risk of incident T2DM, microvascular complications, and all‐cause mortality when compared to bisphosphonates, reinforcing the potential benefits of denosumab in high‐risk populations, such as those with T2DM and osteoporosis [23]. Despite these known advantages, the impact of denosumab adherence on long‐term mortality and renal outcomes of patients with T2DM and osteoporosis remains unclear. Unlike prior large‐scale Taiwanese cohorts that examined kidney disease patients [22] and international hip–fracture registries [24], the current study was the first to evaluate denosumab adherence, renal safety, and mortality, especially in patients who have both T2DM and osteoporosis—a population that faces unique bone‐metabolic perturbations under hyperglycemia. This study aimed to investigate the association among denosumab adherence, changes in kidney function, and all‐cause mortality in this vulnerable population.

## 2 Methods

### 2.1 Database

This retrospective cohort study utilized data from the Kaohsiung Medical University Hospital Research Database (KMUHRD), which contains comprehensive clinical information, including inpatient and outpatient records, laboratory data, and drug dispensing details. The study population comprised patients with T2DM and osteoporosis who initiated denosumab therapy between January 1, 2012, and December 31, 2017. Diagnoses were identified using International Classification of Diseases (ICD)‐9‐CM and ICD‐10‐CM codes. Drug dispensing records included the prescriber information, drug name, dosage, administration route, and prescription duration. Patient confidentiality was ensured by removing all personal identifiers in accordance with the Personal Information Protection Act. Only authorized researchers accessed the data. All‐cause mortality was ascertained through linkage with the National Death Registry, with the causes of death classified by ICD‐10‐CM codes. The Institutional Review Board of Kaohsiung Medical University Hospital approved this study (KMUHIRB‐E(I)‐20210324) and waived the requirement for informed consent. All study procedures complied with the Declaration of Helsinki.

### 2.2 Study Subjects, Comorbidities, and Medications

Eligible participants were patients diagnosed with osteoporosis who began denosumab treatment within the defined study period. The index date was defined as the date of the first administration of denosumab. The exclusion criteria were as follows: diagnosis of type 1 diabetes mellitus, incomplete demographic information, concurrent use of other osteoporosis medications, dialysis treatment, denosumab use for malignancy (XGEVA), or death within 2 years after treatment initiation.

Adherence to denosumab therapy was operationalized in accordance with the established clinical guidelines recommending administration every 6 months to maintain optimal anti‐fracture efficacy of the therapy. Therefore, patients were stratified into two adherence categories according to the number of doses received within the first 24 months following the index date:

- *High‐adherence* (*HA*): It was defined as the receipt of three or four denosumab injections during the 24‐month period. This threshold reflected patients who maintained regular six‐monthly dosing, thereby representing a clinically meaningful adherence associated with the intended therapeutic benefits.
- *Low‐adherence* (*LA*): It was defined as the receipt of only one or two denosumab doses over the same period, indicating suboptimal adherence insufficient to ensure sustained pharmacologic effect and potentially associated with increased risk of adverse outcomes.

This categorization was consistent with prior real‐world investigations on the pharmacotherapy adherence of patients with osteoporosis and enabled a clear clinical interpretation of treatment patterns with respect to outcomes in this high‐risk population [22].

The collected variables included demographic characteristics, fracture history, hip surgery history, Charlson comorbidity index (CCI, based on ICD‐9‐CM/ICD‐10‐CM codes), and concurrent medication use. Renal function was assessed by comparing the average eGFRs for 1 year before and after denosumab initiation and was calculated using the 2021 CKD‐EPI equation for its accuracy and validation across populations. The primary outcome was all‐cause mortality analyzed using the Kaplan–Meier survival curves and Cox proportional hazard models to estimate the crude and adjusted hazard ratios (Table 1).

**TABLE 1:** Baseline characteristics of new denosumab users with type 2 diabetes mellitus.

	Low adherence	High adherence	*p*
	Mean ± SD (*N*, %)	Mean ± SD (*N*, %)	
Case no.	250	286	
Gender			
Female (*N*, %)	190 (76.00%)	233 (81.47%)	0.122
Male (*N*, %)	60 (24.00%)	53 (18.53%)	
Age (Mean ± SD)	77.03 (±9.71)	76.43 (±7.86)	0.435
Age category (*N*, %)			
65−	27 (10.80%)	21 (7.34%)	0.164
65–74	66 (26.40%)	93 (32.52%)	
75+	157 (62.80%)	172 (60.14%)	
History of fracture			
Hip fracture (*N*, %)	91 (36.40%)	109 (38.11%)	0.683
Non‐hip fracture (*N*, %)	37 (14.80%)	49 (17.13%)	0.463
Hip surgery history	112 (44.80%)	126 (44.06%)	0.863
CCI score	2.41 (±2.03)	2.42 (±2.24)	0.950
CCI score category			
2−	101 (40.40%)	125 (43.71%)	0.615
2–3	80 (32.00%)	92 (32.17%)	
4+	69 (27.60%)	69 (24.13%)	
HbA1C	7.08 (±1.50)	6.77 (±1.20)	0.016
Medication			
NSAIDs	70 (28.00%)	89 (31.12%)	0.430
Corticosteroids	64 (25.60%)	64 (22.38%)	0.383
Anticoagulants	61 (24.40%)	61 (21.33%)	0.398
Diuretics	62 (24.80%)	78 (27.27%)	0.516
Antipsychotic	23 (9.20%)	18 (6.29%)	0.207
Thyroxine	4 (1.60%)	9 (3.15%)	0.246
Antihypertensive	82 (32.80%)	98 (34.27%)	0.720
Sedative	38 (15.20%)	43 (15.03%)	0.958

Abbreviations: CCI, Charlson comorbidity index; NSAIDs, non‐steroidal anti‐inflammatory drugs; SD, standard deviation.

### 2.3 Statistical Analysis

All statistical analyses were performed using SAS version 9.4 (SAS Institute, Cary, NC, USA). Categorical and continuous variables were presented as percentages and means ± standard deviations, respectively. Between‐group comparisons were conducted using Student's *t*‐test, paired *t*‐test, and chi‐squared test for independent continuous, dependent continuous, and categorical variables, respectively. A survival analysis was performed using Kaplan–Meier curves for all‐cause mortality. The association between denosumab adherence and mortality was evaluated using univariate and multivariate Cox proportional hazard models. The results were expressed as hazard ratios (HRs) and 95% confidence intervals (CIs). Multivariate models were adjusted for age, gender, and CCI. Statistical significance was defined as *p* < 0.05.

## 3 Results

**FIGURE 1:**
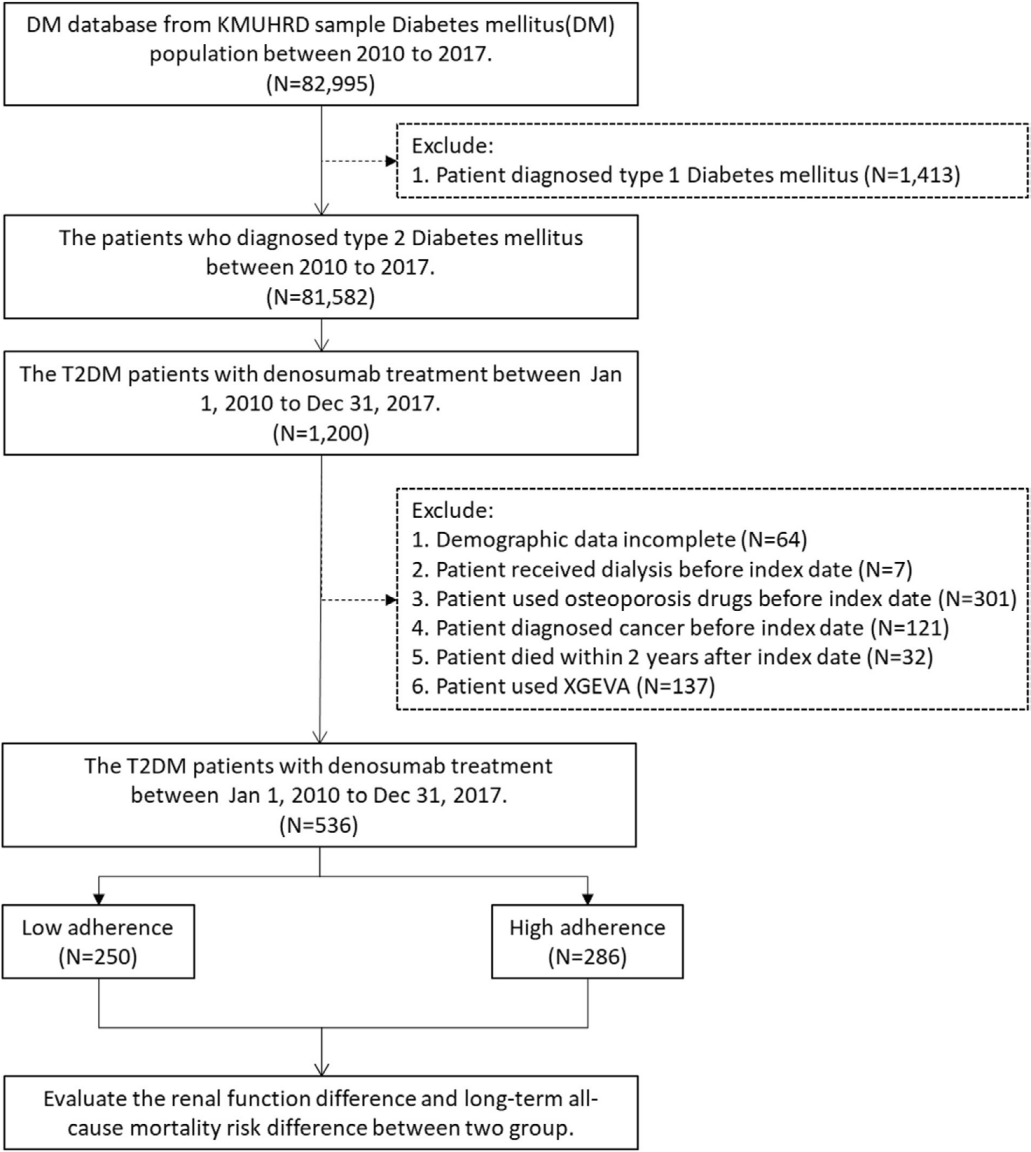
Flowchart of this study. KMUHRD: Kaohsing Medical University Research Database.

From an initial cohort of 81,582 T2DM patients (2010–2017), we identified 536 eligible denosumab users after applying the following exclusion criteria: type 1 diabetes (*n* = 1413), incomplete demographics (*n* = 64), dialysis dependence (*n* = 7), prior osteoporosis medication use (*n* = 301), baseline malignancy (*n* = 121), XGEVA indication (*n* = 137), and early mortality (< 2 years post‐initiation, *n* = 32) (Figure 1).

The final cohort (*n* = 536) was stratified into HA (*n* = 286) and LA groups (*n* = 250). The final cohort had a mean age of 76.7 years, with female predominance (≈80%). Over 60% of patients were elderly (> 75 years), while fewer than 10% were middle‐aged (45–64 years). Both groups (HA and LA) showed comparable comorbidity burdens (mean CCI = 2.4). The HA group exhibited significantly better glycemic control (lower HbA1c) than the LA group (*p* < 0.05). The utilization patterns of geriatric medications, including nonsteroidal anti‐inflammatory drugs, corticosteroids, anticoagulants, diuretics, antipsychotics, thyroid replacement, antihypertensives, and sedatives, were comparable between both groups (Table 1).

**FIGURE 2:**
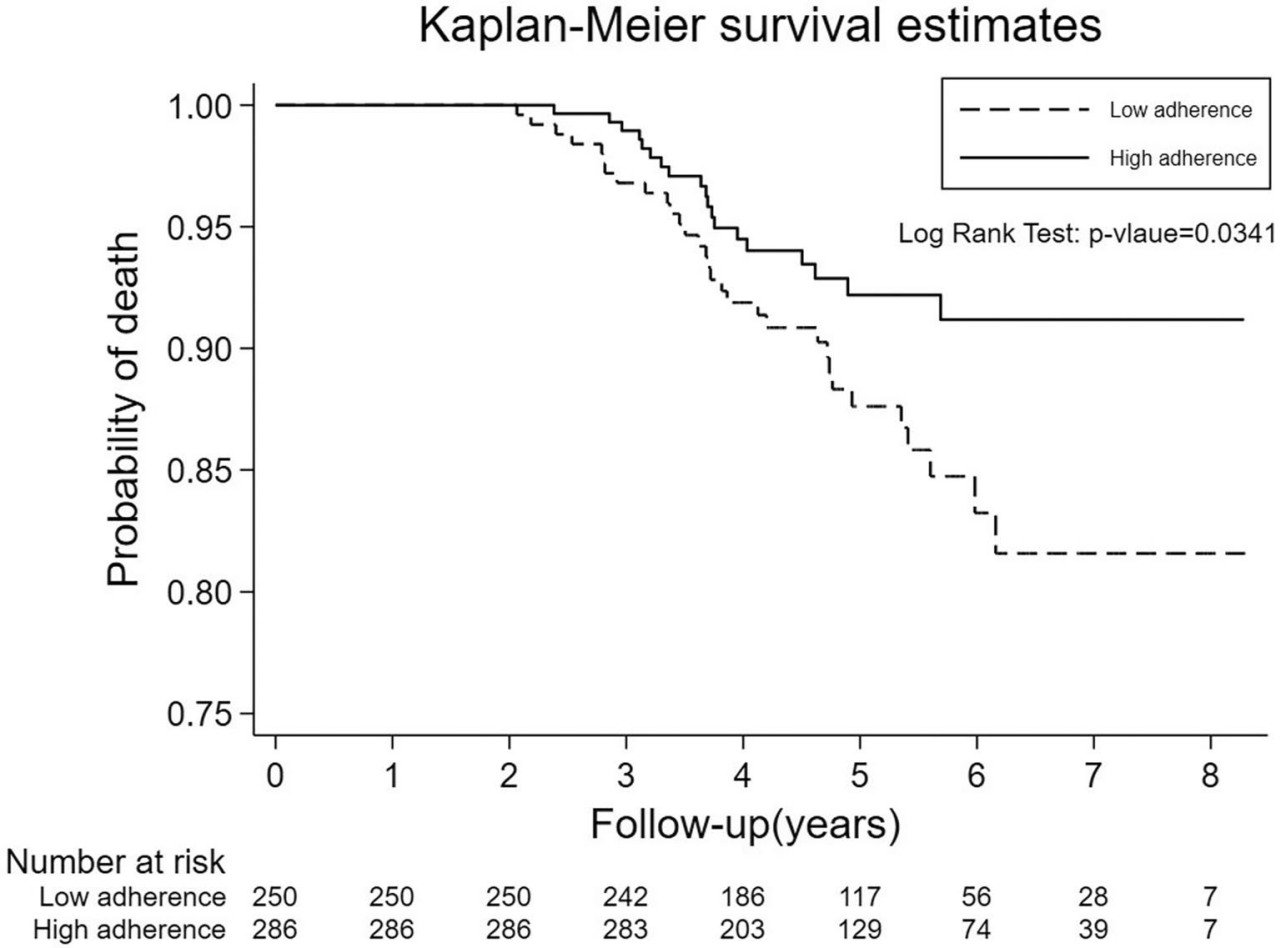
Kaplan–Meier curves for all‐cause mortality, between high‐ and low‐adherence denosumab users with type 2 diabetes mellitus.

The renal function analysis revealed similar rates of eGFR decline in both groups following 1 year of denosumab therapy (ΔeGFR HA: −1.8 vs. LA: −2.1 mL/min/1.73 m^2^, *p* = 0.32) (Table 2). Survival analysis of the HA group exhibited 19 deaths over a total of 1439.8 person‐years, corresponding to a mortality rate of 13.2 per 1000 person‐years. In contrast, the LA group exhibited 31 deaths over 1257.6 person‐years, with a higher mortality rate of 24.65 per 1000 person‐years. The crude HR for all‐cause mortality in the HA group compared to the LA group was 0.54 (95% CI: 0.31–0.93, *p* = 0.037). After adjustment for age, sex, and CCI, the adjusted HR was 0.52 (95% CI: 0.29–0.92, *p* = 0.026), indicating that high adherence was associated with a 48% lower risk of all‐cause mortality compared to low adherence. The Kaplan–Meier survival curves further demonstrated that survival probabilities differed between the groups (log‐rank *p* = 0.034) (Table 3, Figure 2).

**TABLE 2:** Comparison of renal function in patients treated with denosumab in the type 2 DM cohort.

	Low adherence	High adherence	*p* for independent *t*‐test
*N*	127	210	
Pre‐eGFR(mL/min/1.73 m^2^)	54.57 ± 30.40	61.08 ± 26.47	0.040
Post‐eGFR(mL/min/1.73 m^2^)	49.02 ± 29.91	55.54 ± 25.80	0.035
ΔeGFR(mL/min/1.73 m^2^)	5.55 ± 14.17	5.54 ± 13.27	0.994
*p* for Paired samples *t* test	< 0.001	< 0.001	

*Note*: eGFR, estimated glomerular filtration rate; Pre‐eGFR, one‐year average eGFR before the first denosumab treatment; Post‐eGFR, one‐year average eGFR after the first denosumab treatment; ΔeGFR, (pre‐eGFR)–(post‐eGFR).

**TABLE 3:** Crude and adjusted hazard ratios of all‐cause mortality between high‐ and low‐adherence denosumab users.

Groups	*N*	Person‐year (PY)	Mortality rate (per 1000PY)	Cox proportional hazards model			
				Crude		Adjusted	
				HR (95% CI)	*p*	HR (95% CI)	*p*
Low adherence	31 (12.4%)	1257.6	24.65	Ref.		Ref.	
High adherence	19 (6.6%)	1439.8	13.20	0.54 (0.31–0.96)	0.037	0.52 (0.29–0.92)	0.026

Abbreviations: CI, confidence interval; HR, hazard ratio; Ref., reference.

## 4 Discussion

This retrospective cohort study analyzed data from a Taiwanese electronic healthcare database to examine how denosumab adherence is related to the renal function changes and all‐cause mortality in patients with T2DM and osteoporosis. Among 536 eligible patients, those with high adherence experienced a 48% lower risk of mortality (adjusted HR = 0.52, 95% CI: 0.29–0.92), independent of the renal function trajectories. This finding was consistent with the evidence from several nationwide Taiwanese cohort studies. For instance, Yu et al. reported that good adherence to anti‐osteoporosis therapies is linked to reduced post‐hip fracture mortality [18]. Wu et al. further showed that higher adherence was associated with lower all‐cause mortality among denosumab users [22]. Tsai et al. also found that both high adherence and prolonged denosumab treatment were associated with decreased mortality following hip fracture [24].

However, it is important to note the key differences between these studies and the present work. Wu et al. examined denosumab users with CKD but did not stratify patients by diabetic status, while Tsai et al. focused on post‐hip fracture populations rather than on the evaluation of long‐term mortality specifically in patients with T2DM. These distinctions underscore the novel contribution of our analysis in the unique T2DM–osteoporosis subgroup. Additionally, our study was the first to target a metabolically and clinically distinct T2DM–osteoporosis subgroup, demonstrating a substantial survival benefit associated with high denosumab adherence.

Patient‐reported barriers to osteoporosis medication adherence include fear of side effects (53.3%), dislike of taking medications (25.3%), and belief that treatment will not help manage their condition (16.7%) [25]. Denosumab's biannual dosing schedule might address the “dislike of taking medications” barrier, while its established efficacy profile can help address patient concerns about treatment benefits.

Maintaining denosumab adherence is particularly important due to the risk of the rebound phenomenon [26, 27]. Discontinuation or delayed administration of denosumab (e.g., by more than 16 weeks) has been associated with a significantly increased risk of vertebral fractures, which might lead to a higher mortality [26]. Additionally, 1 year after denosumab discontinuation, patients might experience worsened fall risk and sarcopenia measures, further contributing to adverse outcomes [28]. Therefore, clinicians should emphasize the importance of timely denosumab administration, even though denosumab is recognized as the most convenient antiresorptive medication with high patient adherence and persistence [29].

Denosumab's efficacy in increasing BMD and reducing the risk of fracture is well established, primarily through its inhibition of the RANKL‐mediated osteoclast activity [30, 31]. Beyond its effects on the bones, emerging evidence suggests that denosumab might confer cardiovascular and metabolic benefits [32]. Denosumab has been shown to attenuate vascular calcification by potentially inhibiting the RANKL‐mediated osteogenic differentiation of vascular smooth muscle cells and reducing aortic arch calcification [33]. RANKL is implicated in plaque destabilization and thrombosis. Its inhibition might help stabilize atherosclerotic lesions. Tsai et al. also proposed that denosumab might lower parathyroid hormone levels, indirectly reducing cardiovascular risk. However, the clinical significance of this effect requires further investigation [33]. In terms of metabolic outcomes, denosumab is associated with improved insulin sensitivity and glycemic control in patients with T2DM, potentially via its effects on bone‐derived hormones, such as osteocalcin, and by reducing hepatic insulin levels [34, 35]. These additional benefits might partially explain the lower mortality observed in patients with high denosumab adherence; however, further research is needed to validate potential causes.

Furthermore, we found no significant differences in the renal function decline between the HA and LA groups, with both experiencing eGFR reduction after denosumab initiation. This decline was consistent with the natural aging process and the known accelerated decline in patients with T2DM [36, 37]. Denosumab is not metabolized or excreted by the kidneys. In addition, its pharmacokinetics are unaffected by renal function. Multiple clinical trials, including the FREEDOM extension study, have demonstrated that denosumab is equally effective and safe in patients with normal renal function and those with mild to moderate CKD [19, 22]. No significant differences in BMD [38] gains, fracture reduction, or adverse renal outcomes have been observed across the renal function subgroups. In contrast, bisphosphonates are primarily excreted by the kidneys and might accumulate in patients with impaired renal function, increasing the risk of nephrotoxicity and limiting the use of bisphosphonates in cases with advanced CKD. Real‐world data further showed that, unlike bisphosphonates, denosumab does not increase the risk of acute kidney injury or CKD progression, especially in patients under 70 years of age [39].

This study has several limitations that warrant consideration. First, the retrospective observational design limited our ability to establish causality between denosumab adherence and mortality outcomes. The lack of randomization and reliance on existing medical records rendered our results susceptible to unmeasured confounding. The key clinical and socioeconomic variables, including lifestyle behaviors such as smoking and alcohol use, body mass index, BMD, levels of physical activity, and socioeconomic status, were not available in our dataset. Each factor could have influenced both patients' likelihood of adhering to denosumab therapy and the risk of adverse clinical outcomes in these patients.

Although our statistical models adjusted for age, sex, and CCI, several potential confounders could not be captured due to database limitations. We specifically lacked data on calcium and vitamin D supplementation, BMD readings, and use of novel anti‐diabetic medications, such as SGLT2 inhibitors and GLP‐1 receptor agonists. Notably, our study period concluded in 2017. Till then, SGLT2 inhibitors and GLP‐1 receptor agonists had not been widely reimbursed or prescribed in Taiwan. While this limitation reflects contemporary practice patterns during the study window, it leaves room for residual confounding related to medication use.

The use of administrative health databases introduces additional challenges, including potential inaccuracies in coding, underreporting of clinical events, and the inability to validate diagnoses by chart review. For instance, vertebral fractures are often underrepresented because morphometric fractures are seldom coded in claims data, which might result in an underestimation of fracture‐related morbidity and its contribution to mortality risk.

Our efforts to explore the impact of denosumab across different stages of chronic kidney disease were also constrained by the limited number of participants with advanced renal impairment. Consequently, we were unable to conduct robust subgroup analyses across different CKD stages, and our findings may not fully reflect the effects of denosumab in patients with severe renal dysfunction. Larger, multicenter studies are warranted to elucidate these relationships.

We also recognize that our adherence classification—dichotomized as high or low based on the number of denosumab doses received over a 24‐month period—did not capture the full complexity of real‐world medication‐taking behavior. Furthermore, we did not have precise data on the timing of each injection, limiting our ability to assess the consequences of delayed or irregular dosing.

Importantly, our study design might introduce survivor bias because we excluded patients who died within 2 years after starting denosumab administration. This criterion implied that only individuals who survived beyond 2 years were included in the analysis, potentially skewing the observed survival benefit in favor of those already less likely to experience early adverse events of denosumab. Thus, our results might overestimate the association between adherence and long‐term survival compared to a true intention‐to‐treat analysis that includes all patients from treatment initiation.

Our study was conducted at a single academic center in Taiwan, which might have limited generalizability to other populations or healthcare systems, where differences in the cultural, economic, or care delivery factors could affect adherence and outcomes. Several other bias sources remain unaddressed, including the inability to control concomitant medication use, supplementation practices, and missing data on laboratory markers, BMD, and patient‐reported outcomes. These gaps restrict a more nuanced exploration of underlying biological mechanisms, likely contributing to residual confounding.

Finally, we only assessed all‐cause mortality without information on the specific causes of death. This limitation precluded us from determining whether or not survival benefits were attributable to reduced fracture risk, cardiovascular protection, or other mechanisms. The relatively short follow‐up might also have limited our ability to observe the true long‐term effects of sustained denosumab therapy or the risks associated with treatment discontinuation.

In summary, these limitations highlight the need for prospective, multicenter studies with more detailed clinical, laboratory, and patient‐reported data, as well as a longer follow‐up. Such research will be essential for validating our findings and further elucidating the mechanisms by which denosumab adherence might improve outcomes in this high‐risk patient population.

## 5 Conclusion

This study was the first to demonstrate the significant association between denosumab adherence and reduced all‐cause mortality risk, without any adverse effects on renal function, in a T2DM–osteoporosis cohort. Our findings highlighted the dual skeletal and metabolic benefits of denosumab in this high‐risk population, especially among patients with compromised kidney function who might not be able to tolerate bisphosphonates. Our results also emphasize the critical importance of maintaining consistent denosumab treatment adherence to maximize survival benefits and reduce the risk of negative outcomes linked to treatment discontinuation.

Despite these strengths, our study had several limitations. The retrospective and observational design precluded causal inference. In addition, unmeasured confounding factors, such as lifestyle behaviors, bone health metrics, and medication use, may have influenced our results. The study's reliance on administrative data limited the granularity of clinical information, including BMD, bone turnover markers, and cause‐specific mortality. Additionally, the generalizability of our findings may be restricted by the single‐center Taiwanese cohort.

In the future, prospective and multicenter studies with comprehensive clinical, laboratory, and patient‐reported data are warranted to validate our findings, further elucidating the mechanisms linking denosumab adherence to improved outcomes in this high‐risk population. By addressing these gaps, subsequent research can potentially inform clinical strategies to optimize osteoporosis management and reduce mortality in patients with T2DM.

## Ethics Statement

This study complied with the principles of the Declaration of Helsinki. The data used in this study were approved by the Institutional Review Board of the Kaohsiung Medical University Hospital [KMUHIRB‐E(II)‐20210324].

## Conflicts of Interest

The authors declare no conflicts of interest.

## Data Availability

The data that support the findings of this study are available from the corresponding author upon reasonable request.
